# Four-Year Management of Severe COPD–OSA Overlap Syndrome with Phased, Closed-Loop Respiratory Rehabilitation: A Case Report

**DOI:** 10.3390/healthcare14172695

**Published:** 2026-08-24

**Authors:** Qiufeng Peng, Dajun Liu, Hongbo Xue, Haoyang Hei, Hua Guo, Jingchun He, Kui Ye

**Affiliations:** 1 Department of Respiratory and Critical Care Medicine, Tianjin Fourth Central Hospital, Tianjin Medical University, Tianjin 301700, China; pqf6666@163.com (Q.P.);; 2 Department of Vascular Surgery, Tianjin Fourth Central Hospital, Tianjin Medical University, Tianjin 301700, China; 3 Department of Vascular Surgery, Tianjin Fourth Central Hospital, Tianjin University, Tianjin 300072, China

**Keywords:** respiratory rehabilitation, COPD-OSA overlap syndrome, closed-loop management, long-term follow-up, case report

## Abstract

**Background**: Severe COPD–OSA overlap syndrome remains challenging to manage, and existing approaches rarely combine guideline-directed OSA therapy with sustained pulmonary rehabilitation, leaving patients vulnerable to recurrent acute exacerbations. **Case Presentation**: A 71-year-old male with severe overlap syndrome had been hospitalized three times for hypercapnic encephalopathy requiring invasive ventilation. Following admission in 2021, we implemented a four-year, three-stage closed-loop rehabilitation program combined with multimodal OSA management guided by the American Academy of Sleep Medicine (AASM) and 2023 GOLD recommendations. The program included personalized BiPAP titration, quarterly pressure adjustments, positional therapy, and targeted weight control. **Management and Outcomes**: Over four years of follow-up, this combined approach improved arterial blood gas values and exercise tolerance and markedly improved sleep-disordered breathing parameters, with no readmissions for respiratory failure. **Conclusions**: Guideline-conformant multimodal OSA therapy combined with staged closed-loop respiratory rehabilitation may offer an effective long-term strategy for severe overlap syndrome. However, as the findings of a single case report are hypothesis-generating, they require validation in larger prospective studies.

## 1. Introduction

The coexistence of chronic obstructive pulmonary disease (COPD) and obstructive sleep apnea (OSA), collectively termed overlap syndrome (OS), is a complex multimorbid condition with systemic manifestations [1]. Pulmonary rehabilitation—integrating respiratory muscle training, exercise, airway clearance, education, and psychosocial support—is an evidence-based cornerstone of chronic respiratory disease management. Although pulmonary rehabilitation comprehensively addresses symptoms, function, and quality of life, the current evidence base focuses predominantly on COPD, with limited data specific to the OS population [2].

We report a case of COPD–OSA overlap syndrome in a patient who had three prior ICU admissions for hypercapnic encephalopathy requiring invasive ventilation. He was readmitted in July 2021 with an acute exacerbation and fluctuating respiratory failure and was subsequently stabilized through a phased, closed-loop respiratory rehabilitation program. This case provides valuable clinical insights into the long-term integrated management of overlap syndrome. The patient’s clinical course and management are presented below.

## 2. Case Description

A 71-year-old male patient presented to our outpatient clinic with a more than 20-year history of coronary heart disease and hypertension. He had a 20 pack-year smoking history (20 cigarettes per day for 20 years) and had quit smoking 30 years earlier. No significant occupational exposure to dust or toxic substances was reported. The patient had a 30-year history of persistent cough, sputum production, and shortness of breath. He experienced three episodes of acute deterioration with unconsciousness, requiring admission to the Intensive Care Unit (ICU) of Tianjin Fourth Central Hospital in 2014, 2015, and 2017. These episodes were diagnosed as type II respiratory failure with hypercapnic encephalopathy, and all required invasive mechanical ventilation via endotracheal intubation and intensive treatment. Outpatient therapy consisted of irregular use of inhaled salmeterol–fluticasone (50/500 μg) and tiotropium, with poor treatment adherence. His physical condition was severely restricted, as manifested by dyspnea when climbing two flights of stairs and marked limitation in daily activities.

The patient was admitted to the Department of Respiratory and Critical Care Medicine on 29 July 2021, with a chief complaint of frequent cough, sputum production, and wheezing that had worsened over the preceding two weeks. Physical examination revealed an obese (BMI 32 kg/m^2^), conscious patient with cyanotic lips, barrel chest, decreased tactile fremitus, hyper-resonant percussion, coarse breath sounds with wheezing, tachycardia (102 bpm), and a soft, distended abdomen without tenderness or edema.

Arterial blood gas analysis on admission revealed a pH of 7.27, PaCO_2_ of 83 mmHg, and PaO_2_ of 86.4 mmHg. Laboratory tests showed leukocytosis (WBC 11.9 × 10^9^/L) with neutrophilia (78.5%). Chest radiography revealed chronic bronchitis, emphysema, and bilateral interstitial changes. Admission diagnoses included acute exacerbation of COPD (AECOPD) with type II respiratory failure, coronary artery disease (New York Heart Association [NYHA] class IV), and stage 3 hypertension at very high cardiovascular risk (Table 1).

Initial management comprising antibiotics, methylprednisolone, doxofylline, noninvasive ventilation (NIV), and nikethamide was initiated. However, the patient deteriorated rapidly within three hours, with impaired consciousness. Repeat arterial blood gas (ABG) analysis revealed severe respiratory acidosis (pH 7.199, PaCO_2_ 96 mmHg) with preserved oxygenation (PaO_2_ 88.3 mmHg). After excluding other possible causes, type II respiratory failure with hypercapnic encephalopathy was confirmed, and the patient was urgently transferred to the ICU for invasive mechanical ventilation.

Upon return to the respiratory department on 22 August 2021, cough, sputum production, and dyspnea persisted and were severe enough to interfere with lying down, dressing, and walking. Examination revealed a tachypneic patient who was responsive to simple instructions, with conjunctival edema, slight lip cyanosis, a barrel chest with reduced vocal resonance, hyper-resonant percussion notes, coarse breathing with wheezes and rales, a heart rate of 88 bpm, and no lower limb edema. Follow-up chest radiography showed progression of infiltrates in both lower lung zones. Arterial blood gas analysis indicated a pH of 7.315, PaCO_2_ of 81.9 mmHg, and PaO_2_ of 95.5 mmHg.

Treatment was continued with noninvasive ventilation, anti-infective drugs, anti-inflammatory agents, and bronchodilators, together with regular inhalation of salmeterol–fluticasone and tiotropium. Given the patient’s symptoms and obesity, obstructive sleep apnea was suspected. Subsequent polysomnography confirmed severe OSA (apnea–hypopnea index [AHI] = 28 events/h) with severe nocturnal hypoxia (minimum oxygen saturation 68%, T90 > 70%). As no central cause of sleep apnea was identified, central sleep apnea was excluded, confirming the diagnoses of OSA and overlap syndrome (OS).

**Figure 1:**
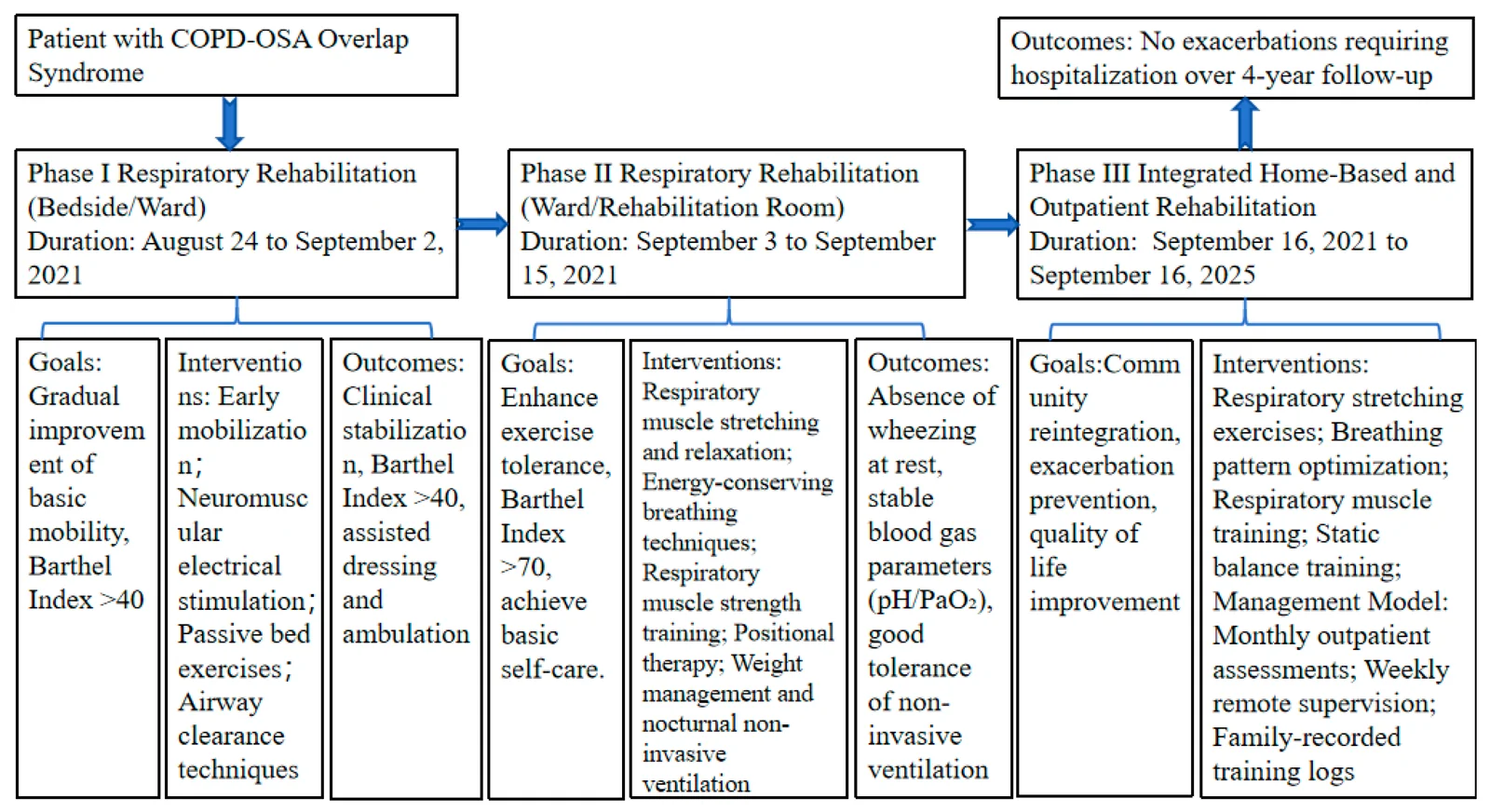
Clinical Pathway of the Three-Phase Respiratory Rehabilitation Program.

**Figure 2:**
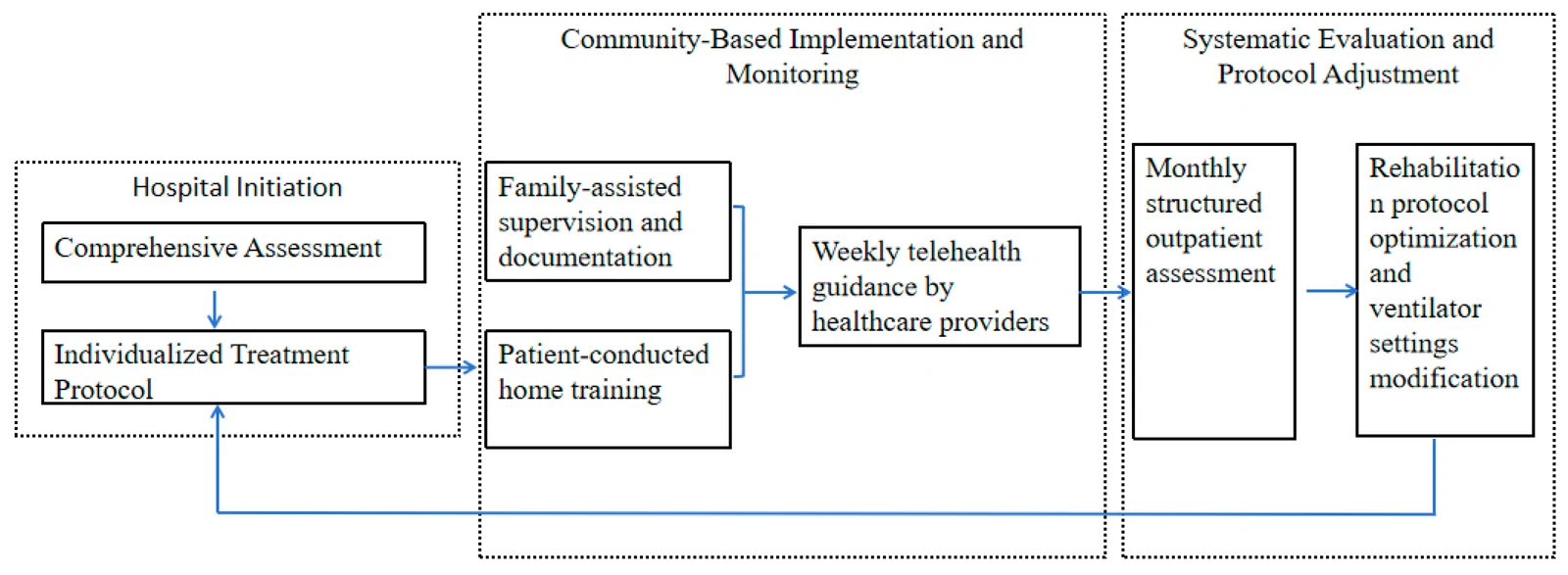
Closed-Loop Long-Term Management Clinical Pathway.

On 24 August 2021, the baseline evaluation showed a COPD Assessment Test (CAT) score of 35 and a modified Medical Research Council (mMRC) dyspnea grade of 4. A stepwise respiratory rehabilitation program was then carried out (Figure 1 and Figure 2).

### 2.1. Phase I Respiratory Rehabilitation (Bedside/Ward), 24 August to 2 September 2021

The intervention included: (1) acute medical treatment; (2) gradual movement from sitting to standing; (3) functional evaluation; (4) daily neuromuscular electrical stimulation (HANS-200A, 10 mA, 10 Hz, 20 min) of the respiratory muscles together with therapist-assisted passive limb exercises (5–8 repetitions per joint); and (5) airway clearance by postural percussion and the directed cough technique (deep inspiration with a 2 s breath hold), performed three times daily (10 min per session).

The objectives of Phase I rehabilitation were to gradually enhance basic mobility (Barthel Index score > 40), to maintain joint movement within normal limits, and to prevent muscle atrophy. The functional aims were for the patient to require assistance with eating, washing, dressing, and walking on level surfaces, to need help with toileting and bathing, to be unable to climb stairs, and to maintain continence.

Intermittent noninvasive ventilation combined with bedside rehabilitation produced notable improvement. The patient’s mental status improved from drowsy and sluggish to awake and oriented. Arterial blood gas values improved (pH 7.34, PaCO_2_ 65 mmHg, PaO_2_ 92 mmHg), sputum clearance improved, and chest radiography showed decreasing infiltration. The Phase I rehabilitation goals were achieved on 2 September 2021, as evidenced by a Barthel Index score > 40, stable arterial blood gases (pH > 7.30, PaCO_2_ < 70 mmHg), improved mental status, and adequate sputum clearance, allowing progression to Phase II.

### 2.2. Phase II Respiratory Rehabilitation (Ward/Rehabilitation Room), 2–15 September 2021

#### 2.2.1. Phase II Rehabilitation Protocol

The structured program included five main components: (1) respiratory muscle stretching and relaxation exercises performed in a standing position, involving slow raising and lowering of the arms with chest expansion during inhalation and abdominal contraction during exhalation (3–5 repetitions), and discontinued if dizziness or palpitations occurred; (2) energy-saving techniques using a forward-leaning posture with upper limb support during episodes of dyspnea; (3) training with threshold loading devices to strengthen the respiratory muscles (5–15 repetitions per session, for at least 3 weeks); (4) sleep positional therapy using the lateral decubitus position; and (5) combined weight control and nocturnal bilevel positive airway pressure (BiPAP) ventilation, with pressure settings (IPAP 12–15 cmH_2_O, EPAP 5–6 cmH_2_O) adjusted according to the patient’s tolerance and nocturnal oxygen saturation. To align with international clinical guidelines for the treatment of OSA, a standardized multimodal intervention plan was incorporated into the Phase II rehabilitation program:

Consistent with the American Academy of Sleep Medicine (AASM) clinical practice guidelines for OSA and the 2023 GOLD recommendations for COPD–overlap syndrome [3,4], this multimodal OSA management regimen was formulated in strict accordance with standardized titration and long-term monitoring criteria. The specific guidelines applied were the AASM’s clinical practice guidelines for the use of positive airway pressure in the treatment of adult obstructive sleep apnea [5], the AASM clinical guidelines for the manual titration of positive airway pressure [4], the 2023 GOLD recommendations for the management of COPD–overlap syndrome [3], and the ATS clinical practice guideline on weight management in adult obstructive sleep apnea [6]. The complete guideline-based OSA intervention framework adopted in this case comprised three core standardized modules:

Polysomnography-guided initial BiPAP titration: In accordance with positive airway pressure (PAP) titration standards, we set a baseline EPAP of 5–6 cmH_2_O to eliminate supine upper airway collapse and titrated IPAP to 12–15 cmH_2_O to suppress apneic and hypercapnic events, maintaining a pressure support difference of 4–10 cmH_2_O as recommended by international ventilation guidelines [4]. Lateral decubitus positional therapy was simultaneously prescribed as a first-line adjuvant measure for positional OSA to reduce supine apnea frequency without additional ventilator pressure escalation [7].

Dynamic quarterly pressure adjustment protocol: After discharge, polysomnography and overnight oximetry were repeated every 3 months during monthly outpatient follow-up. If the patient’s AHI remained above 10 events/h or T90 exceeded 30%, IPAP was gradually increased to 13–16 cmH_2_O and EPAP adjusted to 5–7 cmH_2_O to match long-term disease changes, consistent with the long-term PAP maintenance strategy for overlap syndrome [8].

Guideline-mandated weight management intervention: In accordance with the ATS obesity–OSA treatment guidelines, structured dietary restriction and regular aerobic training were integrated into daily rehabilitation to reduce pharyngeal soft tissue accumulation and upper airway collapsibility, with a target BMI below 28 kg/m^2^ to achieve a sustained reduction in OSA severity [6].

This standardized, guideline-aligned multimodal OSA regimen was not limited to ventilator support; rather, it was an integrated strategy targeting upper airway collapse, nocturnal hypoventilation, and metabolic risk factors.

The objectives of Phase II rehabilitation were to achieve clinical stabilization; to enhance exercise tolerance (Barthel Index score > 70); to perform basic activities independently (eating, washing, dressing, and toileting); to require assistance with bathing and stair climbing; to walk 50 m on level surfaces with assistance; and to maintain continence.

The patient achieved the Phase II objectives, as demonstrated by a Barthel Index score > 70, the ability to walk 50 m with assistance, independence in basic activities of daily living, and stable arterial blood gases (PaCO_2_ < 60 mmHg); he was able to walk short distances without significant breathlessness, thereby meeting the discharge criteria.

#### 2.2.2. Pre-Discharge Assessment (15 September 2021)

At discharge, the patient had no wheezing, and his cough and sputum production had resolved; his mental status, appetite, and sleep were satisfactory. Physical examination showed that he was obese (BMI 32 kg/m^2^) and alert, without cyanosis, with a barrel chest, decreased vocal resonance, hyper-resonant percussion, absent breath sounds, a normal heart rate (82 bpm), and no edema.

Arterial blood gas analysis revealed a pH of 7.346, PaCO_2_ of 56.2 mmHg, and PaO_2_ of 68.9 mmHg. Pulmonary function testing demonstrated an FEV1 of 0.646 L and an FEV1/FVC ratio of 27.1%. In the 6 min walk test (6MWT), the patient walked 179 m (metabolic equivalent of task [MET] 2.59). The CAT score was 30, and the mMRC dyspnea grade was 3.

The patient had no wheezing at rest, a considerable reduction in cough and sputum production, a stable arterial pH close to 7.34, and PaCO_2_ below 60 mmHg; he could dress and walk independently and tolerated noninvasive ventilation well. All these indicators met the discharge criteria, and the patient was discharged on 15 September 2021.

### 2.3. Phase III Respiratory Rehabilitation (Home-Based/Outpatient Integration), September 2021 to September 2025

Phase III commenced upon discharge and continued for four years, combining home-based and outpatient rehabilitation with an emphasis on respiratory stretching exercises, breathing pattern adjustment, and respiratory muscle training; the protocol is detailed in Table 2. During this period, comprehensive assessments—including arterial blood gas analysis, pulmonary function testing, the 6 min walk test, polysomnography, and quality-of-life questionnaires (CAT, mMRC, ESS)—were performed at 6 months, 1 year, and annually thereafter. Monthly outpatient visits and weekly telemonitoring were maintained throughout.

### 2.4. Comprehensive Rehabilitation Protocol

Breathing exercises included diaphragmatic breathing in the supine position with tactile monitoring: one hand was placed on the chest and the other on the abdomen to ensure proper diaphragmatic movement during slow nasal inhalation and controlled exhalation through the mouth. These were combined with pursed-lip breathing to maintain an inspiration-to-expiration ratio of 1:2. Lower limb endurance training involved stationary cycling with an optimally adjusted seat (15° knee flexion at maximum pedal depression) and was performed at a moderate intensity guided by the Borg dyspnea score (2/10) and a target heart rate of 100–110 bpm. Resistance training included upper limb exercises using 0.25 kg dumbbells for multiplanar movements and continuous lower limb strengthening exercises such as squats and heel raises. Balance training included dynamic stepping movements simulating functional activity impairments, as well as single-leg static standing with gradually increasing difficulty. All exercises were performed according to the principles of joint protection and pain-free movement. The program emphasized postural control, coordinated breathing, and gradual progression under supervision during regular treatment sessions. Nocturnal noninvasive ventilation (BiPAP; IPAP 13–16 cmH_2_O, EPAP 5–7 cmH_2_O) was continued together with weight control, with settings adjusted according to the AHI and nocturnal oxygen saturation. Assistance was provided through monthly outpatient visits, weekly telemonitoring via WeChat video calls—during which the patient reported symptoms, oxygen saturation, and training completion, and the rehabilitation team provided real-time feedback and adjusted the home exercise prescription as needed—and family supervision of training records (Table 2).

Rehabilitation objectives included independence in activities of daily living, unrestricted walking, climbing three flights of stairs, community reintegration, prevention of deterioration, and improvement of quality of life and longevity.

After four years of regular rehabilitation and closed-loop management, significant progress was observed; the four-year follow-up data are presented in Table 3.

Respiration and sleep function: Hypercapnia decreased (PaCO_2_ from 56.2 to 35.9 mmHg), reflecting improved ventilatory efficiency. Sleep-disordered breathing improved substantially (AHI from 28 to 2 events/h), with a marked reduction in nocturnal hypoxemia (T90 from 70% to 9%) and resolution of excessive daytime sleepiness (Epworth Sleepiness Scale [ESS] score from 15 at discharge to 6 at four years).

Quality of life and symptoms: Health status improved substantially (CAT from 30 to 12), with sustained mMRC grade 2 dyspnea, indicating clinically meaningful symptom control and functional gain.

Exercise capacity: The 6 min walk distance increased from 179 to 240 m, with enhanced muscle endurance in the chair-stand and arm-curl tests.

Weight management: BMI decreased from 32 to 27.8 kg/m^2^.

Importantly, no hospitalizations for COPD exacerbations occurred during the 4-year follow-up period, and the patient’s range of activities expanded (Table 3).

## 3. Discussion

### 3.1. OSA: Epidemiology, Pathophysiology and Impact on COPD

The prevalence of obstructive sleep apnea (OSA) is increasing, particularly among older adults [9]. OSA results from upper airway obstruction, inflammatory alterations, and abnormal neuromuscular control, causing recurrent airway collapse, hypercapnia, intermittent hypoxia, and sleep disturbance [10]. In patients with chronic obstructive pulmonary disease (COPD), OSA increases the risk of hypercapnia, and the apnea–hypopnea index (AHI) and T90 correlate with disease severity [11]. Recurrent events cause oxygen desaturation, cognitive impairment, and daytime sleepiness; craniofacial abnormalities, obesity, and neural dysfunction are probable contributing factors [12]. Chronic OSA is associated with higher cardiovascular and metabolic risk, and its combination with COPD in overlap syndrome further impairs respiratory muscle function and worsens outcomes [13].

### 3.2. Diagnostic and Management Strategies for OSA and COPD

The diagnosis of OSA integrates clinical manifestations (obesity and snoring) with polysomnographic evidence of obstructive events and requires the exclusion of neurological causes. Management includes pharmacotherapy, ventilatory support (e.g., BiPAP), and individually tailored lifestyle modifications. Surgical intervention (e.g., tonsillectomy or airway reconstruction) is reserved for anatomical abnormalities or refractory cases. For COPD management, the GOLD 2023 recommendations include initiation of pulmonary rehabilitation during acute exacerbations and moderate-to-high-intensity aerobic training for stable disease to improve VO2max [6]. A multicenter trial demonstrated that 1-year home-based exercise enhanced sit-to-stand performance and self-perceived health in COPD patients, and maintenance rehabilitation is advised for COPD with sleep-breathing disorders [13].

### 3.3. Multidimensional Benefits of Pulmonary Rehabilitation in OSA

Unlike previous short-term rehabilitation studies that focused only on COPD training, this four-year closed-loop model incorporates comprehensive, guideline-aligned multimodal intervention as the main supportive therapy. Pulmonary rehabilitation can improve respiratory function, cardiopulmonary fitness, psychological state, sleep quality, and self-management while reducing complications [14]. Specific breathing techniques enhance diaphragmatic movement, alveolar ventilation, and respiratory muscle function, whereas combined aerobic and strength training improves physical capacity, lung function, dyspnea, and sleep quality while reducing apnea frequency, hospitalization rates, and mortality [15]. Psychological support and education improve disease understanding and reduce anxiety and depression; personalized programs should therefore integrate respiratory training, physical fitness training, nutrition, and behavioral strategies [15]. Educating patients on self-monitoring (e.g., blood oxygen saturation and symptom recording) can promote engagement, whereas frail patients require comprehensive assessment to ensure safe and individualized rehabilitation [16].

A multicenter prospective trial in patients with stable chronic obstructive pulmonary disease (COPD) indicated that patients with severe COPD could gain greater benefits from endurance or resistance training after one year [17]. However, that study focused on short-term interventions and did not address long-term closed-loop management after severe acute exacerbations of COPD. Our case describes a comprehensive long-term closed-loop respiratory rehabilitation program in an elderly, obese, former-smoking male with a long medical history, frailty, multiple comorbidities, and frequent acute exacerbations of overlap syndrome (OS). He had a history of type II respiratory failure and had been admitted to the ICU three times for hypercapnic encephalopathy, each time requiring invasive mechanical ventilation, indicating that his condition was extremely severe.

His breathing difficulty reflected persistent, severe disease, with a sustained baseline arterial partial pressure of carbon dioxide (PaCO_2_) of 83 mmHg that was associated with OSA. This chronic condition places sustained stress on the respiratory muscles, causing fatigue and further worsening pulmonary function impairment. Pulmonary function testing showed a forced expiratory volume in the first second (FEV1) of 0.646 L and an FEV1/FVC ratio of 27.1%. As the patient had both COPD and OSA, the relative contribution of each disease to the ventilatory dysfunction could not be determined. His daytime sleepiness and cognitive impairment (ESS 15 at discharge) limited daily activities and social interactions; he experienced shortness of breath after climbing one flight of stairs, and dressing and moving were also difficult. OSA posed a significant cardiovascular threat in this patient: during treatment, he experienced an episode of ventricular tachycardia/fibrillation with hypotension (Adams–Stokes syndrome), consistent with the cardiovascular risk of overlap syndrome in patients with pre-existing coronary disease.

The patient had a history of inconsistent use of inhaled medications and was initially reluctant to participate in rehabilitation. To enhance compliance, we implemented two complementary strategies: (1) strengthened communication with the patient and his family, explaining the treatment rationale and drawing on his military background for motivation; and (2) a phased, personalized rehabilitation program with progressively increasing difficulty, regular check-ups, and goal-directed feedback. These strategies improved his engagement, self-management, and long-term adherence. This three-stage model (inpatient, outpatient, and home-based), combined with remote telemonitoring and monthly structured assessments, offers a framework for sustained disease management. Over the four years, compliance rose in parallel with symptomatic and functional improvement.

Breathing exercises (e.g., diaphragmatic and pursed-lip breathing) improve diaphragmatic excursion, alveolar ventilation, and ventilatory efficiency and reduce nocturnal hypoventilation and apneas [18]. Pursed-lip breathing increases airway pressure during expiration, preventing airway collapse and improving ventilation. Aerobic and resistance exercises likewise increase physical capacity and lung function and reduce dyspnea, with favorable effects on sleep apnea severity.

Regular physical activity contributed to weight reduction, with BMI declining from 32 kg/m^2^ to 27.8 kg/m^2^, and reduced upper airway fat deposition and collapsibility, consequently diminishing apnea severity. Weight loss and exercise enhance sleep continuity and autonomic function [19]. The standardized OSA management strategy formulated in accordance with AASM and GOLD guidelines likely contributed, together with the three-stage closed-loop respiratory rehabilitation, to the sustained, multidimensional clinical improvements observed over the four-year follow-up, which may explain this patient’s favorable long-term outcomes:

Because the AOS management and rehabilitation programs were implemented simultaneously, it is not possible to fully separate the contribution of each component to the observed outcomes in this single patient. However, improvements in specific AOS parameters (AHI from 28 to 2 events/h, T90 from 70% to 9%, minimum SpO2 from 68% to 88%, ESS from 15 to 6, and resting PaCO_2_ from 56.2 to 35.9 mmHg) are best explained by the OSA treatment component, which directly targets upper airway collapse and reduced ventilation during sleep. The gains in exercise capacity (6MWT from 179 to 240 m), muscle strength, and CAT score (from 30 to 12), on the other hand, are more likely related to the rehabilitation component. As this is a single case report, these associations should be interpreted with caution.

First, targeted BiPAP ventilation combined with positional therapy was associated with a marked reduction in recurrent nocturnal upper airway obstruction: the AHI decreased from 28 to 2 events/h and T90 from 70% to 9%. Together with diaphragmatic and pursed-lip breathing training, which improved alveolar ventilatory efficiency, this regimen may have contributed to the gradual resolution of the patient’s chronic hypercapnia, with resting PaCO_2_ declining from 56.2 mmHg at discharge to 35.9 mmHg after four years and nocturnal hypoxemia substantially improved. These findings are consistent with cohort evidence that standardized PAP therapy improves gas exchange in patients with overlap syndrome [8].

Second, long-term guideline-based weight control combined with aerobic and strength rehabilitation training appeared to benefit both respiratory muscle function and OSA severity. A reduction in BMI may have decreased extrathoracic impediments and reduced fat deposition in the throat, while gradual training of the respiratory muscles may have diminished respiratory muscle fatigue caused by intermittent nocturnal hypoxia. Over the four years, the patient’s FEV1 remained stable, the six-minute walk distance progressively increased, and mMRC grade 2 dyspnea was maintained without further deterioration [6].

Third, the integration of OSA ventilatory correction with outpatient–home closed-loop rehabilitation may have addressed the main trigger of the patient’s recurrent hypercapnic encephalopathy. No further ICU admissions occurred during the four-year follow-up, which is consistent with clinical evidence that standardized PAP therapy for OSA reduces the severity of COPD exacerbations and the risk of readmission in patients with overlap syndrome [20].

Without long-term OSA management, nocturnal hypoventilation would likely persist and predispose the patient to hypercapnic decompensation, even after routine pulmonary rehabilitation.

Fourth, with formal OSA treatment, nocturnal sleep fragmentation and chronic intermittent hypoxia improved, and excessive daytime sleepiness markedly decreased (ESS from 15 to 6). Concurrently, progressive rehabilitation enhanced daily mobility and self-care ability, lowering the CAT score from 30 to 12 and substantially improving overall quality of life.

The modest FEV1 recovery, while not dramatic, may reflect improved respiratory muscle endurance rather than reversal of fixed obstruction.

Decreases in CAT and mMRC grades suggested better symptom control. Blood gas parameters (lower PaCO_2_, higher PaO_2_) indicated improved gas exchange and ventilatory efficiency. The reduction in AHI from 28 to 2 events/h, the rise in minimum SpO2, and the fall in T90 from 70% to 9% all suggested substantial improvement in sleep-disordered breathing.

After four years of this systematic program, the patient showed considerable improvement in compliance and quality of life, expressing satisfaction with both the results and the management approach.

### 3.4. Limitations and Future Perspectives

This case illustrates the potential of comprehensive pulmonary rehabilitation for severe overlap syndrome. However, findings from a single case report cannot be extrapolated to broader populations, and the observations presented here are hypothesis-generating, requiring validation in larger prospective studies. The four-year follow-up did not systematically assess psychological or cardiovascular endpoints, and the observed improvements may partly reflect medication effects, noninvasive ventilation, or the natural disease course. The success of this program also depended on specific institutional resources (monthly polysomnography, dedicated rehabilitation therapists, and a WeChat-based telemonitoring infrastructure) that may not be available in all settings. We cannot exclude a Hawthorne effect from the structured monthly contact, which may have motivated compliance independently of the rehabilitation protocol itself. Whether this model is cost-effective in resource-limited settings remains an open question. Further prospective randomized trials are needed to establish causality and to evaluate cost-effective telerehabilitation approaches.

## 4. Conclusions

In severe COPD–OSA overlap syndrome characterized by frequent exacerbations and repeated ICU admissions, combining guideline-directed multimodal OSA therapy with stepwise inpatient-to-home closed-loop rehabilitation may help sustain clinical stability over several years. Personalized BiPAP titration, positional therapy, weight management, and graded rehabilitation collectively improved gas exchange, sleep-disordered breathing, exercise capacity, and quality of life, with no COPD-related readmissions over four years. Although these findings are hypothesis-generating and require validation in larger prospective studies, this approach provides a practical framework for the long-term management of severe overlap syndrome.

## Figures and Tables

**Table 1:** Baseline Clinical Characteristics of the Patient.

Category	Parameter	Value
Demographics	Age	71 years
	Sex	Male
	Body Mass Index (BMI)	32 kg/m^2^
	Previous Occupation	Military Veteran
Medical History and Comorbidities	Primary Diagnoses	COPD (30-year history), OSA
	Comorbidities	Coronary artery disease, Hypertension (>20 years)
	History of Severe Exacerbations	3 ICU admissions for type II respiratory failure with hypercapnic encephalopathy, requiring invasive ventilation (2014, 2015, 2017)
Personal History and Behavior	Smoking History	20 pack-years (20 cigarettes/day for 20 years), quit 30 years ago
	Pre-admission Medications	Inhaled salmeterol-fluticasone (50/500 μg), Tiotropium powder (irregular use)
	Treatment Adherence (Pre-admission)	Poor (irregular use of inhalers)
Key Admission Physiological Parameters	Arterial Blood Gas (on oxygen)	
	pH	7.27
	Arterial Partial Pressure of Carbon Dioxide (PaCO_2_)	83 mmHg
	Arterial Partial Pressure of Oxygen (PaO_2_)	86.4 mmHg
	Pulmonary Function	
	Forced Expiratory Volume in 1 s (FEV1)	0.646 L
	FEV1/Forced Vital Capacity (FVC)	27.1%
	Polysomnography (at diagnosis)	
	Apnea-Hypopnea Index (AHI)	28 events/h
	Minimum Oxygen Saturation	68%
	Time with Oxygen Saturation < 90% (T90)	70%

**Table 2:** Phase III Pulmonary Rehabilitation Protocol.

	Aerobic Exercise	Resistance Training	Flexibility and Balance
F-frequency	3 sessions/week	3 sessions/week	Daily
I-intensity	Borg dyspnea scale: 2 (mild), exertion: 13–14 (somewhat hard), Target HR: 100–110 bpm	Moderate intensity (30–60% 1RM)	Pain-free full range of motion
T-time	10–20 min	15–20 min	30 s hold per joint, 3 repetitions
T-type	Lower-body cycle ergometry	0.25 kg dumbbells	Static and dynamic balance exercises Volume
V-volume	40–60 min	8–10 muscle groups, 8–12 reps/set, 3 sets to fatigue	

**Table 3:** Four-Year Follow-up Data.

	At Discharge	6 Months Post-Discharge	1 Year Post-Discharge	2 Years Post-Discharge	3 Years Post-Discharge	4 Years Post-Discharge
Pulmonary Function						
FEV1 (L)	0.646	0.652	0.678	0.712	0.701	0.692
FEV1/FVC	27.10%	28.10%	28.90%	29.10%	28.70%	29.20%
Arterial Blood Gas (on Room Air)						
pH	7.34	7.37	7.40	7.37	7.41	7.38
PaCO_2_ (mmHg)	56.2	53.5	44.2	41.8	38.3	35.9
PaO_2_ (mmHg)	68.9	80.7	80.7	82.3	81.7	88.5
Sleep and Metabolism						
BMI (kg/m^2^)	32	30.2	28.9	26.5	27.3	27.8
AHI	28	25	15	16	5	2
Minimum Oxygen Saturation	68%	72%	81%	85%	86%	88%
T90	70%	55%	37%	29%	16%	9%
Quality of Life and Symptoms						
ESS	15	10	8	8	9	6
CAT score	30	20	13	15	15	12
mMRC grade	3	2	2	2	2	2
Exercise Capacity						
6MWT	179	204	210	270	260	240
Metabolic Equivalent (MET)	2.59	2.75	2.79	3.19	3.05	2.99
Cardiorespiratory Fitness Assessment						
30 s Chair Stand Test (Repetitions)	5	7	8	10	8	9
30 s Arm Flexion Test (Repetitions)	6	9	7	8	10	8
Seated Forward Reach Test (Left/Right: cm)	−8/−7	−6/−10	−9.5/−8.5	−6/−8	−6/−8	−5/−6
Back Scratch Test (Left/Right: cm)	−31/−32	−24/−23	−26/−30	−23/−26	−25/−28	−25/−24
Single-Leg Stance Balance Test (Left/Right: Seconds)	8/3	10/3	8/4	7/3	8/4	9/3

FEV1, forced expiratory volume in 1 s; FVC, forced vital capacity; mMRC, modified Medical Research Council dyspnea scale; MET, metabolic equivalent of task; CAT, COPD Assessment Test; BMI, body mass index; ESS, Epworth Sleepiness Scale; AHI, apnea–hypopnea index; 6MWT, 6 min walk test; T90, percentage of total sleep time with oxygen saturation below 90%.

## Data Availability

All data supporting the results of this case report are included within the article and its tables. No additional unpublished data are available.
